# Estimation of spatio-temporal parameters of gait from magneto-inertial measurement units: multicenter validation among Parkinson, mildly cognitively impaired and healthy older adults

**DOI:** 10.1186/s12938-018-0488-2

**Published:** 2018-05-09

**Authors:** Matilde Bertoli, Andrea Cereatti, Diana Trojaniello, Laura Avanzino, Elisa Pelosin, Silvia Del Din, Lynn Rochester, Pieter Ginis, Esther M. J. Bekkers, Anat Mirelman, Jeffrey M. Hausdorff, Ugo Della Croce

**Affiliations:** 1 Department of Biomedical Sciences, University of Sassari, Sassari, Italy; 2 Interuniversity Centre of Bioengineering of the Human Neuromusculoskeletal System, Sassari, Italy; 3 Department of Electronics and Telecommunications, Politecnico di Torino, Turin, Italy; 4 E-services for Life and Health, San Raffaele Hospital, Milan, Italy; 5 Department of Experimental Medicine, Section of Human Physiology and Centro Polifunzionale di Scienze Motorie, University of Genoa, Genoa, Italy; 6 Department of Neuroscience, Rehabilitation, Ophthalmology, Genetics and Maternal Child Health, University of Genoa, Genoa, Italy; 7 Institute of Neuroscience/Newcastle University Institute for Ageing, Clinical Ageing Research Unit, Campus for Ageing and Vitality, Newcastle University, Newcastle, UK; 8 Newcastle Upon Tyne Hospitals NHS Foundation Trust, Newcastle, UK; 9 Department of Rehabilitation Sciences, Neuromotor Rehabilitation Research Group, KU Leuven, Louvain, Belgium; 10 Department of Neurology, Donders Institute for Brain, Cognition and Behaviour, Parkinson Centre Nijmegen, Radboud University Medical Centre, Nijmegen, The Netherlands; 11 Center for the Study of Movement, Cognition and Mobility, Neurological Institute, Tel Aviv Sourasky Medical Center, Tel Aviv, Israel; 12 Sagol School of Neuroscience and Sackler School of Medicine, Tel Aviv University, Tel Aviv, Israel; 13 Rush Alzheimer’s Disease Center and Department of Orthopaedic Surgery, Rush University Medical Center, Tel Aviv, Israel

**Keywords:** Clinical gait analysis, Spatial and temporal gait parameters, Magneto-inertial sensors, Wearable sensors, Parkinson, Elderly, Multicentric study

## Abstract

**Background:**

The use of miniaturized magneto-inertial measurement units (MIMUs) allows for an objective evaluation of gait and a quantitative assessment of clinical outcomes. Spatial and temporal parameters are generally recognized as key metrics for characterizing gait. Although several methods for their estimate have been proposed, a thorough error analysis across different pathologies, multiple clinical centers and on large sample size is still missing. The aim of this study was to apply a previously presented method for the estimate of spatio-temporal parameters, named Trusted Events and Acceleration Direct and Reverse Integration along the direction of Progression (TEADRIP), on a large cohort (236 patients) including Parkinson, mildly cognitively impaired and healthy older adults collected in four clinical centers. Data were collected during straight-line gait, at normal and fast walking speed, by attaching two MIMUs just above the ankles. The parameters stride, step, stance and swing durations, as well as stride length and gait velocity, were estimated for each gait cycle. The TEADRIP performance was validated against data from an instrumented mat.

**Results:**

Limits of agreements computed between the TEADRIP estimates and the reference values from the instrumented mat were − 27 to 27 ms for Stride Time, − 68 to 44 ms for Stance Time, − 31 to 31 ms for Step Time and − 67 to 52 mm for Stride Length. For each clinical center, the mean absolute errors averaged across subjects for the estimation of temporal parameters ranged between 1 and 4%, being on average less than 3% (< 30 ms). Stride length mean absolute errors were on average 2% (≈ 25 mm). Error comparisons across centers did not show any significant difference. Significant error differences were found exclusively for stride and step durations between healthy elderly and Parkinsonian subjects, and for the stride length between walking speeds.

**Conclusions:**

The TEADRIP method was effectively validated on a large number of healthy and pathological subjects recorded in four different clinical centers. Results showed that the spatio-temporal parameters estimation errors were consistent with those previously found on smaller population samples in a single center. The combination of robustness and range of applicability suggests the use of the TEADRIP as a suitable MIMU-based method for gait spatio-temporal parameter estimate in the routine clinical use. The present paper was awarded the “SIAMOC Best Methodological Paper 2017”.

## Background

Instrumented gait analysis provides objective and reliable measures of locomotion patterns and their variability. These measures can contribute to the investigation of gait pathologies and to the definition of a targeted rehabilitation program [1, 2]. Gait analysis is emerging as an effective tool to detect an incipient neurodegenerative disease or to monitor its progression [3, 4]. It has been shown that gait disturbances are an early indicator for mild cognitive impairment (MCI) and can predict progression from MCI to Alzheimer’s disease [5]. Furthermore, gait performance is also a predictor of fall status [6, 7], morbidity and mortality [8, 9].

Objective measures of the temporal and spatial parameters of gait allow to define the level of impairment and to characterize functional gait performance, which can serve as a biomarker of mobility [4, 6, 10]. The computation of the spatio-temporal parameters requires, for each gait cycle, the identification of specific gait events (GEs). These are the initial contact (IC) and final contact (FC) of the foot with the ground.

The most commonly used temporal gait parameters include stride and step duration and cadence. In addition, spatial gait parameters can be defined from the distance covered between two consecutive ICs (step and stride length). Gait spatio-temporal parameters can be estimated from measurements obtained using various sensing technologies, such as foot-switches, inertial sensors, pressure mats, or stereo-photogrammetric systems. While the latter two technologies are relatively expensive and require a controlled and dedicated environment, lengthy set-up and post-processing time, the other two options are comparatively inexpensive and easy to use. In particular, magneto-inertial measurement units (MIMUs) have been frequently presented as an affordable solution to assess gait parameters in a variety of environments [4, 6, 11, 12]. However, the accuracy of the gait spatio-temporal parameters obtained using MIMUs can vary remarkably depending on the algorithms used to detect ICs and FCs and estimate distances [13]. Moreover, methods developed and validated on healthy gait are not guaranteed to be effective in assessing parameters for specific pathological gaits [14]. So far, no study addressed the robustness of the detection algorithm across data coming from multiple clinical centers, despite its value for further supporting clinical use. Finally, and probably most importantly, the majority of the studies in the literature validated MIMU-based methods for the estimation of the gait spatio-temporal parameters only on limited sample sizes [14–19].

A promising method for the automatic GEs detection and spatio-temporal parameters was presented by Trojaniello et al. [14] and tested in real life settings in successive work by Storm et al. [20]. The method, here named TEADRIP (Trusted Events and Acceleration Direct and Reverse Integration along the direction of Progression), was validated on four different gait conditions (i.e. healthy elderly, hemiparetic, Parkinson and choreic gait) and two different walking speeds, and it was shown that its performance was comparable or better than other methods proposed [20, 21].

The aim of the present study was to further extend TEADRIP validation for the spatio-temporal parameters estimation to gait inertial data recorded in a multicenter trial (four clinical centers) on a very large sample size of participants (236) including patients with Parkinson’s disease (PD), MCI and healthy older adults.

## Methods

### Subjects

Two-hundred-thirty-six community-living older adults who self-reported two or more falls within the previous 6 months were enrolled in the study across four clinical centers in four countries (Belgium, Israel, Italy, and the UK). The subjects were part of the randomized controlled trial performed within the EU funded V-Time project and the study was approved by the medical ethics review committee at each site [22]. Eligible individuals were enrolled if they were aged 60–90 years, on stable medication for the past month and able to walk for at least 5 min unassisted (refer to Mirelman et al. [23] for additional eligibility criteria). Individuals who agreed to participate in the study were asked to sign informed written consent. Participants were divided into three groups: older adults with no cognitive impairment (ELD), older adults with mild cognitive impairment (MCI) and people with Parkinson’s disease (PD). Population characteristics for each clinical center are detailed in Table 1.

**Table 1:** Subject characteristics for clinical centers

Clinical center	N	Females	Males	Age Mean ± SD (years)	ELD	PD	MCI
UNIGE	52	35	17	73 ± 5	16	28	8
KULEU	58	40	18	74 ± 7	27	14	17
TASMC	75	37	38	73 ± 7	20	53	2
NEWCA	51	26	25	74 ± 8	17	30	4
Total	236	138	98	74 ± 7	80	125	31

*N* total number, *ELD* healthy older adults, *PD* Parkinson’s disease subjects, *MCI* mild cognitive impaired subjects. (Subjects between centers were age matched)

### Instrumentation

**Fig. 1:**
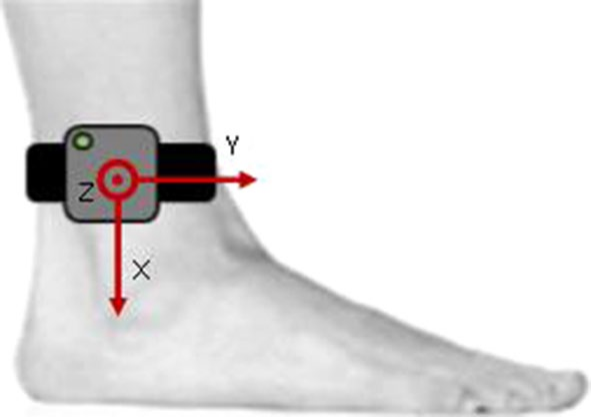
Sensor placement (R-MIMU) and its local coordinate system axes

Two synchronized MIMUs (Opal, APDM Inc), featuring a tri-axial accelerometer, gyroscope and magnetometer (unit weight 22 g, unit size 48.5 mm × 36.5 mm × 13.5 mm) were used. Inertial data were streamed wirelessly to a laptop (“robust synchronized streaming mode”) and stored for offline analysis. Sampling frequency was set at 128 Hz and the accelerometer range at ± 6 g. The MIMUs were attached with velcro straps to the subject ankles, laterally, about 30 mm above the malleoli. The sensors were aligned approximately along the three anatomical directions with X, Y and Z axes pointing downward, forward and to the right, respectively, for the MIMU on the right ankle (R-MIMU), and downward, backward and to the left for the MIMU on the left ankle (L-MIMU) (Fig. 1).

An estimate of the MIMUs local coordinate system (LCS) orientation with respect to the global coordinate system (GCS) was provided by the manufacturer’s proprietary software. A spot check of the MIMU performance was performed according to the guidelines proposed previously [24]. The GEs and spatio-temporal parameters resulting from the processing of the recordings of an instrumented 7-m instrumented mat acquiring data at 120 Hz (Zeno Walkway, ProtoKinetics LLC) and analyzed with a dedicated software (PKMAS, ProtoKinetics LLC) were used for validation purposes. The instrumented mat measurements had a temporal accuracy of ± 1 sample (about 8 ms) and spatial resolution accuracy of ± 12.7 mm. The MIMU and the instrumented mat were synchronized via hardware (~ 8 ms). A custom-made cable was used to apply an external trigger generated by the instrumented mat to the access point controlling the MIMUs.

### Experimental protocol

The data acquisition took place in the following laboratories: the Center for the Study of Movement, Cognition, and Mobility, Tel Aviv Sourasky Medical Centre, Israel (TASMC); the Neuromotor Rehabilitation Research Group, KU Leuven, Belgium (KULEU); the Clinical Ageing Research Unit, Newcastle University and Newcastle upon Tyne Hospitals NHS Foundation Trust, UK (NEWCA); the laboratory of the Department of Neurosciences, University of Genoa, Italy (UNIGE).

Recordings started with subjects standing still for a few seconds at 3 m from the instrumented mat and then walking back and forth for about 1 min at a comfortable speed (normally paced walk, NW) along a 12-m walkway which included the instrumented mat in its central portion. The same protocol was repeated at a higher walking speed (fast paced walk, FW). Subjects wore their own shoes and they could rest in between acquisitions if needed. Walking aids such as canes or tripods were allowed if used in daily life.

### Gait events identification and gait temporal and spatial parameters estimation

A preliminary analysis was performed to eliminate operator-dependent swap between right and left MIMUs.

A first approximate segmentation of MIMU signals into gait cycles was performed by detecting the peaks in the medio-lateral (Z) component of the angular velocity. These peaks usually occur during the leg swing motion. Gait cycles not detected or erroneously detected in this processing phase lead to missed or extra GEs, respectively.

**Fig. 2:**
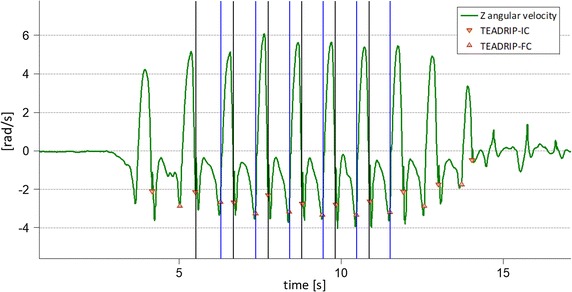
TEADRIP and instrumented mat gait events. GEs for the first passage over the mat (right side only). GEs identified by the TEADRIP method are depicted as red triangles, while GEs identified from the instrumented mat are depicted as vertical lines (black IC, blue FC)

Both ICs and FCs were then identified as in [14], although the FC search interval was made to begin at the minimum Z angular velocity rather than the maximum Y acceleration, being the former easier to identify. An example of IC and FC identification during a passage on the instrumented mat is depicted in Fig. 2. Once the ICs and FCs were identified from both R-MIMU and L-MIMU signals, the following gait temporal parameters were calculated per gait cycle for both sides: Stride Time, Step Time, Swing Time and Stance Time.

The stride length was also estimated as described by Trojaniello et al. [14]. For each stride, ankle acceleration components were expressed in the GCS and, after gravity removal, optimally filtered and direct and reverse integrated (OFDRI technique [25]). The direction of progression was found by rotating the axes on the horizontal plane until one component of the velocity resulting from the above-mentioned integration was maximized. The MIMU acceleration was reoriented accordingly. The acceleration component along the direction of progression was integrated by means of the OFDRI, using as initial integration value the MIMU estimated forward linear velocity, given by the product of the Z angular velocity at mid-stance and the MIMU distance from the malleolus [26]. A further simple integration provided the forward displacement during a stride cycle (Stride Length). Gait Velocity was calculated for each cycle as Stride Length divided by Stride Time.

Temporal and spatial parameters resulting from TEADRIP were discarded when a stride was not fully included in the instrumented mat. Spatial parameters were discarded when the estimate of the MIMU GCS orientation as provided by the manufacturer’s software failed. In case of freezing of gait for the PD subjects, the relevant portion of the trial was excluded from the analysis.

### Errors associated to the gait events identification and spatio-temporal parameters estimation

To estimate the accuracy of the TEADRIP method, only gait data recorded while the participant walked on the instrumented mat (straight walking without turns) were considered. This gait data selection was made by excluding, for each passage over the mat, MIMU data recorded before the first IC and after the last FC as identified by the instrumented mat.

A GEs matching procedure was implemented to ensure that an unexpected additional time delay between MIMUs and instrumented mat would not compromise the comparison of their outputs. To match a TEADRIP estimated IC with the corresponding IC measured with the instrumented mat, a search interval around the latter was defined, which spanned from the FC preceding the IC to the FC following the IC. The TEADRIP estimated IC that fell in the interval was selected as the matching IC. If more than one TEADRIP estimated IC was found in the search interval, the farthest from the IC measured by the instrumented mat was counted as an extra IC, while if none fell in the interval a missed IC was counted. If an extra TEADRIP estimated IC was found between two subsequent mat-measured FCs further apart than 1.3 s (which is approximately the average higher limit for PD stride duration, [27]), then the entire gait cycle was discarded (mat measure failure). The same procedure was applied to match TEADRIP estimated FCs to the corresponding FCs measured by the instrumented mat.

For each gait cycle, the stride-by-stride errors affecting the TEADRIP estimations of the GEs and the spatio-temporal parameters were computed as differences with respect to the relevant measurements obtained from the instrumented mat. Difference plots (Bland–Altman) were used to visually check the distributions of the spatio-temporal parameters errors between the two measurement systems.

For each subject, the mean error (*me*) and mean absolute error (*mae*) values for the estimated GEs and gait spatio-temporal parameters were calculated by averaging stride-by-stride errors computed over the entire gait trial (left and right sides were not differentiated). The standard deviation of the stride-by-stride error (*sde*) was also determined for each recorded trial. The TEADRIP estimations of the gait temporal and spatial parameters were also evaluated using the ratio between the *mae* and the mean value of the parameter as measured by the instrumented mat (*%mae*).

A three-way repeated measures analysis of variance (ANOVA) was performed on the *mae* for both GEs and spatio-temporal parameters to investigate the difference in the errors between subject groups (ELD, MCI, PD), between clinical centers (UNIGE, KULEU, TASMC, NEWCA) and within imposed walking speed (NW, FW). Since GEs *mae* were found not to be normally distributed (as resulted from a Shapiro–Wilk test), they were transformed to a logarithmic scale in order to ensure a normal distribution before undergoing ANOVA. Where a significant difference was found, post hoc tests for subject groups and clinical centers were performed with Bonferroni correction. All data was analyzed using SPSS v.24 (IBM Corporation) at a 5% level of significance.

## Results

Over 15,000 gait cycles (see Table 2) were selected from the instrumented mat and compared to those identified using the TEADRIP.

**Table 2:** Number of initial contacs and strides analyzed in each clinical center

Clinical center	Initial contacts	Stride Time estimates	Stride Length estimates
UNIGE	5818	3512	3387
KULEU	5405	4156	4072
TASMC	7168	5824	5759
NEWCA	3632	2636	2585
Total	22,068	16,167	15,840

The number of Stride Length estimates differs from that of Stride Time since stride length values were not computed for those trials in which the estimate of the MIMU GCS orientation failed

The mean and standard deviation values of the mean trial values of the spatio-temporal parameters as determined by the instrumented mat in each clinical center at the two gait speeds are reported in Table 3.

**Table 3:** Gait spatio-temporal parameters mean values (sd) across subjects for clinical centers and walking speeds, as measured from the instrumented mat

Clinical center	Stride Time (s)		Stance Time (s)		Swing Time (s)		Step Time (s)		Stride Length (m)		Gait Velocity (m/s)	
	NW	FW	NW	FW	NW	FW	NW	FW	NW	FW	NW	FW
UNIGE	1.09 (0.09)	0.99 (0.09)	0.72 (0.07)	0.64 (0.07)	0.38 (0.03)	0.36 (0.03)	0.55 (0.04)	0.50 (0.05)	1.11 (0.16)	1.21 (0.16)	1.02 (0.17)	1.23 (0.21)
KULEU	1.13 (0.20)	1.02 (0.16)	0.73 (0.18)	0.64 (0.14)	0.40 (0.04)	0.38 (0.03)	0.57 (0.10)	0.51 (0.08)	1.19 (0.21)	1.31 (0.24)	1.09 (0.29)	1.33 (0.33)
TASMC	1.13 (0.14)	1.00 (0.13)	0.74 (0.11)	0.64 (0.10)	0.40 (0.07)	0.36 (0.04)	0.56 (0.07)	0.50 (0.07)	1.12 (0.25)	1.25 (0.23)	1.01 (0.26)	1.27 (0.29)
NEWCA	1.09 (0.08)	0.98 (0.10)	0.71 (0.07)	0.63 (0.08)	0.38 (0.03)	0.35 (0.03)	0.54 (0.04)	0.49 (0.05)	1.16 (0.19)	1.26 (0.23)	1.07 (0.20)	1.30 (0.29)

*NW* normal paced trials, *FW* fast paced trials

### Gait event identification and spatio-temporal parameters estimation errors

**Fig. 3:**
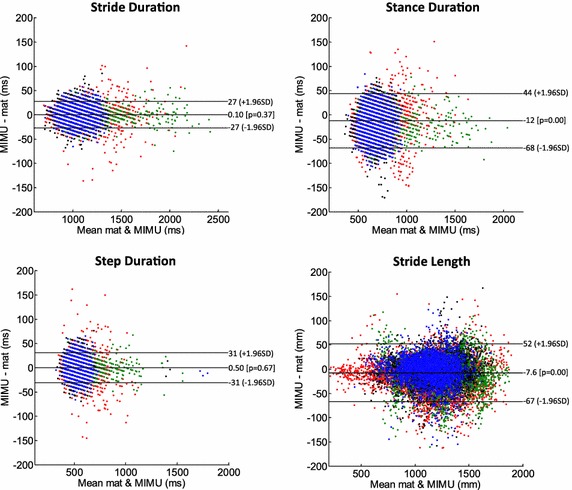
Difference (Bland–Altman) plots for stride, stance and step durations and for stride length. Limits of agreement are, respectively, 27, 56, 31 ms and 60 mm. Red: TASMC; green: KULEU; black: NEWCA; blue: UNIGE

The difference plots of Stride, Stance and Step Time and Stride Length are reported in Fig. 3. The estimated limits of agreement were 27 ms (2.6%) for Stride Time, 56 ms (8.5%) for Stance Time, 31 ms (5.8%) for Step Time and 60 mm (5.3%) for Stride Length.

The values of the $\overline{me}$, $\overline{sde}$, $\overline{mae}$ for IC and FC, averaged across the subjects of each clinical center, are reported for both for NW and FW trials in Table 4. The GEs errors for the participants from Newcastle could not be assessed due to a non-constant delay between MIMUs and instrumented mat signals across data acquisition sessions. However, being the delay constant within any acquisition session, this did not affect the estimation of the errors related to temporal parameters. The same descriptive statistics in addition to the $\overline{\% mae}$ are presented in Table 5 for each clinical center (both for NW and FW trials) for Stride Time, Stance Time, Swing Time, Step Time, Stride Length and Gait Velocity. Table 6 reports the subjects *mae* averaged across each group for both NW and FW trials.

**Table 4:** Subject mean error, standard deviation and mean absolute error averaged across clinical centers for both walking speeds (gait events)

Parameter	Clinical center	$\overline{me}$		$\overline{sde}$		$\overline{mae}$	
		NW	FW	NW	FW	NW	FW
Initial contact (ms)	UNIGE	9	9	10	11	15	14
	KULEU	3	4	9	9	11	10
	TASMC	5	8	10	11	12	13
	NEWCA	n.a.	n.a.	n.a.	n.a.	n.a.	n.a.
Final contact (ms)	UNIGE	− 9	− 9	13	13	20	20
	KULEU	− 8	− 7	12	14	21	19
	TASMC	− 3	− 2	12	14	19	17
	NEWCA	n.a.	n.a.	n.a.	n.a.	n.a.	n.a.

$\overline{me}$: subject mean error averaged across centers; $\overline{sde}$: subject error standard deviation averaged across centers; $\overline{mae}$: subject mean absolute error averaged across centers

**Table 5:** Subject mean error, standard deviation, mean absolute error and its relative percentage averaged across clinical centers for both walking speeds (spatio-temporal parameters)

Parameter	Clinical center	$\overline{me}$		$\overline{sde}$		$\overline{mae}$		$\overline{mae \% }$	
		NW	FW	NW	FW	NW	FW	NW	FW
Stride Time (ms)	UNIGE	< 1	< 1	15	14	12	11	1	1
	KULEU	< 1	< 1	12	11	9	9	1	1
	TASMC	< 1	< 1	14	13	10	10	1	1
	NEWCA	− 1	< 1	15	15	12	11	1	1
Stance Time (ms)	UNIGE	− 20	− 18	17	17	29	27	3	3
	KULEU	− 11	− 11	17	15	25	22	2	2
	TASMC	− 8	− 10	17	16	24	23	2	2
	NEWCA	− 11	− 12	17	15	24	23	3	4
Swing Time (ms)	UNIGE	20	18	17	17	29	27	3	3
	KULEU	11	11	17	16	25	23	2	2
	TASMC	8	10	18	16	24	23	2	2
	NEWCA	12	13	17	15	25	23	2	3
Step Time (ms)	UNIGE	< 1	< 1	16	15	13	12	1	1
	KULEU	< 1	< 1	14	13	11	11	1	1
	TASMC	< 1	< 1	16	15	12	12	1	1
	NEWCA	< 1	< 1	15	14	13	12	2	2
Stride Length (mm)	UNIGE	− 1	− 3	22	27	21	22	2	2
	KULEU	− 8	− 5	19	21	22	25	2	2
	TASMC	− 14	− 15	19	22	26	28	2	2
	NEWCA	− 4	− 6	19	30	19	27	2	2
Gait Velocity (mm/s)	UNIGE	− 2	− 4	23	30	21	24	2	2
	KULEU	− 7	− 5	20	25	21	27	2	2
	TASMC	− 13	− 16	20	25	25	30	3	2
	NEWCA	− 4	− 5	18	36	19	31	2	2

$\overline{me}$: subject mean error averaged across centers; $\overline{sde}$: subject error standard deviation averaged across centers; $\overline{mae}$: subject mean absolute error averaged across centers; $\overline{\% mae}$: mean absolute error referred to parameter estimate averaged across centers

**Table 6:** Group average of the subjects mean absolute errors for the gait events and spatio-temporal parameters for both walking speeds

Parameter	ELD		MCI		PD	
	NW	FW	NW	FW	NW	FW
Initial contact (ms)	10	11	10	10	14	14
Final contact (ms)	21	20	20	19	20	18
Stride Time (ms)	10	9	10	10	11	11
Stance Time (ms)	24	23	23	23	26	24
Swing Time (ms)	24	23	23	23	27	25
Step Time (ms)	11	10	11	10	13	12
Stride Length (mm)	21	25	19	23	23	25
Gait Velocity (mm/s)	21	29	18	25	22	28

*ELD* healthy older adults, *PD* Parkinson’s disease subjects, *MCI* mild cognitive impaired subjects

Table 7 summarizes the ANOVA results; significant differences are indicated in italic. The analysis across clinical centers for the GE errors were performed only between UNIGE, TASMC and KULEU since NEWCA GE errors were not available.

**Table 7:** ANOVA results for the errors in determining the gait events and the gait spatio-temporal parameters

	Initial contact	Final contact	Stride Time	Stance Time	Swing Time	Step Time	Stride Length	Gait Velocity
Walking speed								
F value	0.12	3.30	0.10	1.78	1.59	2.93	4.78	27.32
p value	0.73	0.07	0.76	0.18	0.21	0.09	*0.03*	*0.00*
Clinical center								
F value	1.97	0.56	2.40	0.60	0.50	0.53	1.66	1.59
p value	0.14	0.57	0.07	0.61	0.68	0.66	0.18	0.19
Subject group								
F value	5.21	0.64	3.61	0.81	1.02	4.61	0.01	0.13
p value	*0.01* ^*a*^	0.53	*0.03* ^*b*^	0.45	0.36	*0.01* ^*c*^	0.99	0.88

Significant post hoc results: ^a^ELD-PD (p = 0.01); ^b^ELD-PD (p = 0.01); ^c^ELD-PD (p = 0.01). Underlined results are from the comparison of UNIGE, TASMC and KULEU only

A significant group main effect was found for IC identification. Post hoc analyses revealed that for IC errors there was a significant difference between ELD and PD (p = 0.01), with larger errors for the PD group.

While no temporal parameter error showed any center effect, Stride Time and Step Time errors were significantly different across groups. Post hoc analyses revealed that there was a significant difference for errors between ELD and PD (p = 0.01 for Stride Time and p = 0.01 for Step Time), with larger errors for the PD group.

Group did not have a significant effect on the error of spatial parameters, while there was a significant effect for walking speed.

## Discussion

The tested method was successfully applied on a total of more than 20,000 ICs and FCs collected on 236 older adults (healthy, Parkinsonian and MCI participants). In performing the validation, additional care had to be taken to deal with limitations of the instrumented mat measurements used as reference values for the TEADRIP estimations of the gait parameters, such as steps outside the instrumented surface and unexpected failures.

The average values of the spatio-temporal parameters estimated by the instrumented mat showed a homogeneity across the clinical centers and values consistent with the literature.

The IC $\overline{me}$ showed, in all centers and at both walking speeds, an average delay of up to 10 ms as identified by TEADRIP with respect to that identified by the instrumented mat, while the opposite holds for the FC. The amplitude of the subjects $\overline{sde}$ was slightly higher for the FC confirming the higher uncertainty in detecting FCs as opposed to ICs encountered in most validation studies. Similar conclusions can be drawn by looking just at the $\overline{mae}$ values. The opposite delays for IC and FC TEADRIP estimates reflected in a slight underestimation of the stance phase and an overestimation of the swing phase, but did not have any detrimental effect on the estimation of either Stride Time or Step Time, which showed extremely low $\overline{me}$ values. All temporal parameters exhibited a $\overline{sde}$ for each clinical center between 10 and 20 ms, confirming a limited variability of the errors within the trials at both walking speeds and in all clinical centers. The spatial parameters $\overline{me}$ in all clinical centers and for both walking speeds showed a global slight underestimation performed by TEADRIP. Overall, the $\overline{\% mae}$ of both temporal and spatial parameters was often below and, except NEWCA Stance Time at FW, never over 3% which is an excellent result, although a thorough comparison with the results obtained in studies proposing other methods is not straightforward [1, 15–17, 28–34]. Regarding the estimation of the spatial parameters, it has been shown in the study conducted by Hannink et al. [35], that the OFDRI technique was the best performing among the double integration methods for mobile gait analysis tested in their study.

Even more importantly, all results of TEADRIP estimations were extremely consistent across all clinical centers and with the previous results obtained in a single center on much smaller population samples [14]. Since the *mae*, as opposed to the *me*, is not affected by a potential cancellation due to cycle-differences of opposite signs, it was chosen as the quantity to investigate with the ANOVA, which showed minimal statistical difference in the performance of the TEADRIP across subject groups, clinical centers and gait speeds. In particular, only spatial parameters errors were significantly different between walking speeds. The difference is probably the result of a more difficult estimation of a correct initial constant value needed to estimate velocity from acceleration when the task is performed at higher speed.

Consistently with the results of the previous study employing TEADRIP [14], estimates of ICs for PD subjects were affected by errors significantly different from those obtained in the ELD subject group. In partial disagreement with the results of the previous study, a different error between ELD and PD was also found for Stride Time and Step Time estimations. However, this difference may be a consequence of the above mentioned difference between IC timing errors. These results therefore provide a clear insight of the margin of tolerance associated to the estimation of the different temporal parameters for different populations. For instance, when estimating the IC, an average uncertainty error of 10 ms is expected for ELD and MCI subjects, while slightly higher errors (14 ms) should be considered when analyzing PD subjects.

Overall, the results obtained in this study extend the validity of the TEADRIP method, originally employed in [14] on four smaller subject groups, and combined with the findings of the work of Storm et al. [20], who applied the same gait parameter estimation method to free-living gait, make TEADRIP a well-validated gait parameter estimation method.

## Conclusions

TEADRIP, the gait parameter estimation method employed in this study, was effectively validated on a large number of subjects recorded in four different clinical centers. Not only was the performance comparable to that of the instrumented mat used as a reference, but it was also characterized by a greater amount of recorded data (longer and more diversified walks can be instrumented). Furthermore, as demonstrated in earlier work [20], these results hold also for outdoor straight line walking. The TEADRIP is therefore a valuable candidate for becoming a standard for the estimation of gait spatio-temporal parameters with MIMUs placed on the ankles.

## Abbreviations

MIMU: magneto-inertial measurement unit
PD: Parkinson’s disease
MCI: mild cognitive impairment
ELD: elderly (older adults)
GE: gait events
IC: initial contact
FC: final contact
LCS: local coordinate system
GCS: global coordinate system
NW: normally paced walk
FW: fast paced walk
UNIGE: University of Genova
KULEU: KU Leuven
TASMC: Tel Aviv Sourasky Medical Center
NEWCA: Newcastle University
OFDRI: optimally filtered and direct and reverse integration
TEADRIP: Trusted Events and Acceleration Direct and Reverse Integration along the direction of Progression
